# Development of hydrophobic paper substrates using silane and sol–gel based processes and deriving the best coating technique using machine learning strategies

**DOI:** 10.1038/s41598-021-90855-7

**Published:** 2021-05-31

**Authors:** Kapil Manoharan, Mohd. Tahir Anwar, Shantanu Bhattacharya

**Affiliations:** 1 Microsystems Fabrication Lab, Department of Mechanical Engineering, Indian Institute of Technology Kanpur, Kanpur, India; 2 BNCET, Lucknow, India

**Keywords:** Mechanical engineering, Surface chemistry, Characterization and analytical techniques, Design, synthesis and processing

## Abstract

Low energy surface coatings have found wide range of applications for generating hydrophobic and superhydrophobic surfaces. Most of the studies have been related to use of a single coating material over a single substrate or using a single technique. The degree of hydrophobicity is highly dependent on fabrication processes as well as materials being coated and as such warrants a high-level study using experimental optimization leading to the evaluation of the parametric behavior of coatings and their application techniques. Also, a single platform or system which can predict the required set of parameters for generating hydrophobic surface of required nature for given substrate is of requirement. This work applies the powerful machine learning algorithms (Levenberg Marquardt using Gauss Newton and Gradient methods) to evaluate the various processes affecting the anti-wetting behavior of coated printable paper substrates with the capability to predict the most optimized method of coating and materials that may lead to a desirable surface contact angle. The major application techniques used for this study pertain to dip coating, spray coating, spin coating and inkjet printing and silane and sol–gel base coating materials.

## Introduction

Surfaces with anti-wetting properties with a contact angle of 150° and above find a lot of application in multiple areas of engineering and technology such as automotive and aerospace^1–3^, microfluidics^4^, electronics^5,6^, oil water separation^7,8^ etc. Also it has wide applications in coating currency notes to prevent their wear and tear due to sweating etc.^9,10^.

**Figure 1:**
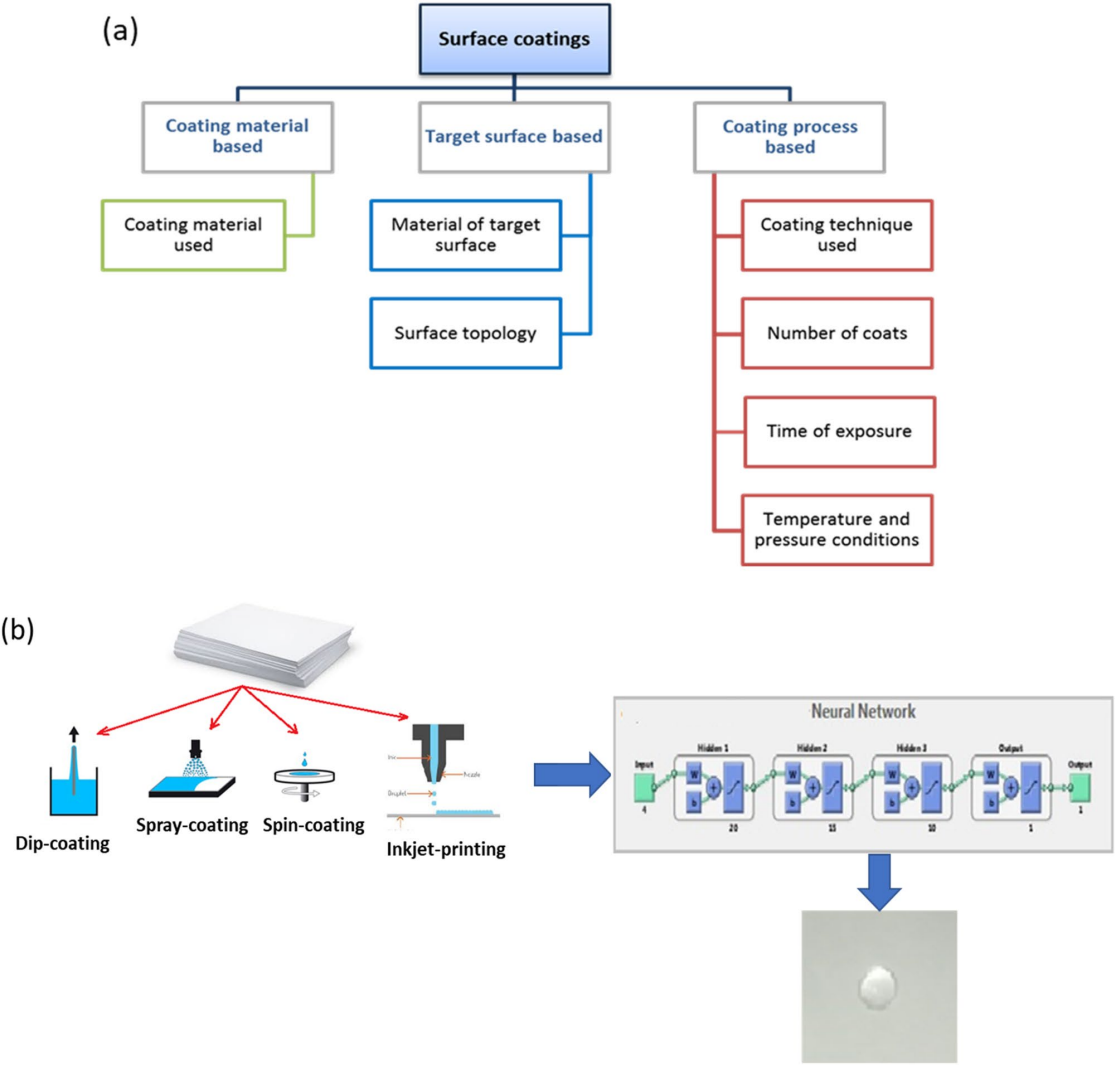
(**a**) Criteria’s and sub-criteria’s which decide the nature of the surface coating, (**b**) Scheme to develop the best coating technique and derive the best set of parameters using machine learning (ML) algorithm.

Deposition of low surface energy materials on selected surfaces like silanes and sol gels^11–17^ especially silica based materials have found prominence due to the ability to self-assemble over selected surfaces^18^. In respect of the properties that may be useful to define the surface energy of any coated surface there can be a broad classification of involved parameters into three independent variables such as (a) parameters relating to the material of the coat, (b) properties related to the surface being coated and (c) further processes used for coating various surfaces^8,18,19^. Figure 1a shows this classification while delving into some sub-criteria’s of the three broad ranging domains.

The coating material domain encompasses various properties like initial viscosity of the material, the density and surface tension of the coating solution and the specific reactions occurring within leading to a change in any of these sub criterions.

Also, not all materials are compatible with all surfaces and they need to be selected based on various properties of the target surface. Some properties include surface roughness, texture etc. Once these coating materials and target surfaces are decided it is important to define various processes which include coating techniques (dip, spin, spray, inkjet printing etc.), number of coats to reach the optimum desired nature, desired temperature and pressure conditions where the surface may be evaluated and also coated and process related parameters for the target process like coating time in processes like dip coating, Fromm number in Inkjet Printing, or spin speeds and surface pretreatment in spin coating, or spray time and viscosity of coating material in spray coating etc.

Earlier research on development of hydrophobic surfaces using low surface energy coatings have been aimed at process development with detailed characterization of various parameters and conditions. Spin and dip coating methods have been used frequently to deposit fluoro-silanes as surface modifiers over different substrates to develop surfaces with contact angle greater than 140°^20,21^. Spray coating techniques have been deployed by Li et. al. for preparing coatings over aluminum substrates which report contact angles of about 156° using fluorinated acrylic random copolymer mixture^22^*.* Latthe and Chouiki have studied coating and printing techniques of inorganic sol–gel over glass surfaces^23,24^. The various process-wise classification of the different material, coating process and process para-metrics are summarized in table S1^19–25^ of supplementary information for obtaining various contact angle ranges.

Although a lot of research have been carried out on the use of materials as mentioned above over several metallic, glassy, textile or silicon surfaces or their derivatives using multiple processes^25–30^, very less work has really been reported on paper substrates to develop surfaces of a suitable hydrophobic nature. Also, there is a gap in the comparative study on how the process of coating, the material used for coating and other relevant conditions influence the anti-wetting properties.

In this study, we have shown process development schemes to produce hydrophobic surface over cellulose based printable papers using various processes and coating materials and use a comparative method to find out the most optimum combination (Fig. 1b). A silane-based ink using TMMS and a sol gel-based ink using TEOS have been developed and used to study the effect of coating materials and number of coating layers on the resulting surface energy of the paper substrate. A comparative study of the relative efficacies of dip coating and inkjet printing processes over spin and spray coating techniques have also been evaluated. The experimental results have been analyzed using neural network and process optimization is carried out to predict outcomes for the desired surface hydrophobicity. The scheme projected through this work in a way shows how to predict a desirable combination of materials of coat, materials of substrate, coating processes and process parameters to yield a desirable surface hydrophobicity level for paper substrates.

## Materials and methods

### Materials

Precursor’s tetraethyl-orthosilicate (TEOS) for sol–gel solution and trimethoxymethylsilane (TMMS) for silane solution as well as ethanol absolute (100% purity), liquor ammonia, cyclohexanol, 2, 6-dimethyl-4-heptanol, hexylene glycol and a-terpineol have been procured (M/s Sigma Aldrich, India). Hydrochloric acid (0.1 M) was procured (M/c CDH Fine Chemicals, India).

### Synthesis: Solution and ink formulation

Initially, 0.5 mL of liquor ammonia and 1 mL of DI water were mixed with 25 mL of ethanol (absolute). The DI water and ammonia serve as accelerating agent and catalyst respectively for the hydrolysis reaction. This solution has been further stirred for 5 min at 150 rpm at room temperature conditions. Further, 100 mL of TMMS has been added to the mix in a dropwise manner and stirred for 10 min at 150 rpm resulting in the final TMMS based silane solution (hereafter called “Ts1”).

For developing the TEOS based sol–gel solution, 10 mL of TEOS has been rigorously mixed with absolute ethanol for 10 min with addition of 1.7 mL, 0.1 M HCL in a dropwise manner in room temperature condition with stirring at 60 rpm for a time period of 6 h resulting in a sol–gel solution (hereafter called “Ts2”) with a solid content of 14% (by weight).

The as prepared solutions are not enabled to be used directly for inkjet printing as they do not conform to the viscosity and surface tension requirements posed by the printing devices. The Fromm no. for good printability for all inks have to be optimized between 4 and 14 which is a need in this case as well^31^. Specific agents are further added to these inks to counter any evaporation due to changes in environmental conditions or processing conditions. Supplementary material, Figure S2, shows different steps for processing of each of the solutions and ink as derived through processing so far. Cyclohexanol and 2, 6 Dimethyl-4-Heptanol are used as viscosity and surface tension controlling agents in order to bring desirability of relevant parameters for printing. Hexylene glycol has been used as a solvent and ᾰ-terpineol has been added for evaporation control in the TEOS process.

For the TMMS process Cyclohexanol and 2,6 Dimethyl-4-Heptanol are added to ‘Ts1’ in desired proportions and stirred appropriately to obtain silane-based ink (hereafter called ‘Ti1’). The ‘Ts2’ is further evaporated to obtain a solution having 50 wt. % to which ᾰ-terpineol is added stirred and heated to evaporate the residual ethanol, DI water and HCl respectively. The final solution in this case is viscous and transparent and Hexylene glycol, Cyclohexanol and 2, 6 Dimethyl-4-Heptanol are respectively added and stirred in proportions to get desirable printing parameters (hereafter known as ‘Ti2’). The complete ink formulation flow chart showing process details is available in supplementary Figure S2. Both the inks are developed with simultaneous viscosity and surface tension measurements to obtain the desirable Fromm number.

### Coating process and techniques

For coating the solutions, the first step has been to check the effect of both ‘Ti1’ and ‘Ti2’ on paper substrates. Cellulosic paper strips are prepared and vertically dipped in 5 mL solution of each kind for 60 secs followed by slow retraction (1 mm/s) and drying for 30 min at STP. Similar set of experiments are done for Ts1 and Ts2 solution samples for comparison. Just to recall, the Ti1 and Ti2 are controlled parametrically to the order of printing whereas Ts1 and Ts2 are not tailored to the printing parameters. These trials provided a fairly good estimate of relative changes due to coating various solutions over paper substrates.

Further the effect of various coating processes and process parameters are studied and optimized. The various process-wise classification of the different material, coating process and process parametric are summarized in Table 1 for obtaining various contact angle ranges. All experiments are carried out at 25 °C unless specified.

**Table 1:** Coating experiment process parametric and values.

S.No	Parameters		Values
1	Substrate	Cellulosic paper	20 mm x 50 mm (Surface area: 1000mm^2^)
2	Coating Material	TEOS based Sol ink (Ti1)	Viscosity: 0.0109 ± 0.0005 Pa.s, Surface tension: 30.59 ± 0.5 N/m
		TMMS based Silane ink (Ti2*)*	Viscosity: 0.0114 ± 0.0006 Pa.s, Surface tension: 29.43 ± 0.45 N/m
3	Coating Processes	Dip coating	Dip time: 30, 60, 90, 120 sec Retraction speeds: 1 mm/sec Vertically dipped
		Spin Coating	Spin speeds: 150, 300 rpm
		Spray Coating	Spray speed: 5, 10, 15 mL/min
		Inkjet Printing	Low quality print (20 prints/ mL) High quality print (10 prints/ mL)
4	Coating Cycles	Number of dips, spin, spray or prints	Number of cycles: 7, Drying time: 30 min

Dip coating is carried out using a program-controlled dip coater mechanism for the various dipping times and retraction speeds. SPS150 manual dispensing spin coater is used for spin coating while a pneumatic controlled sprayer is used for the spraying experimentation. An EPSON L130 inkjet printer is used for printing freshly prepared ink with the printing properties set for different printing qualities.

### Characterization

Viscosity of all the prepared solutions Ts1, Ts2 and inks Ti1 and Ti2 were measured at 25 °C with an Anton Paar Rheometer. Additionally, the surface tension was also measured through the plate method using a fully automatic Surface Tension meter (K100, M/s KRÜSS GmbH Co., Hamburg, Germany). These measurements are done for the solutions and inks for freshly prepared as well as week old samples to observe the aging behavior.

X-ray diffraction has been further carried out using Pan Analytical X’pert Powder Diffractometer to analyze the chemical changes to the paper surface with coats of various ink solutions. A scan step size of 0.02 in the range of 2θ (20–70°) at 45 kV and tube current 40 mA has been used. It is further analyzed using X’Pert Highscore software to understand the different diffraction peaks of the coated surfaces. Also, the change in peak intensity for different samples have been recorded to understand the change in crystallinity aspects of the coated surface and also the surface energy.

Contact angle goniometer (DATA Physics OCA 15 EC) is used to measure the advancing contact angle of the water droplet using Sessile drop method through a drop of volume 5 μl at room temperature after a drop stabilization time of 10 secs. The SEM images of the coated surface are captured using EVO 50 (CARL Zeiss) system. The contact angle for each of the surfaces is checked at multiple points and the average data is reported with an error of ± 1°.

### Machine Learning (ML) based system

The results recorded using experimental protocols have been used as training samples for the AI based Aritificial neural network (ANN) system that has been developed using MATLAB (MATLAB R2016a). A hybrid optimization technique deploying Gradient descendent (GD) and Gauss–Newton (GN) approaches called as “The Levenberg–Marquardt algorithm (LMA)” has been used to arrive at the most optimal solution for a given set of input parameters/ criteria. Rapid convergence in the vicinity of minimum error as in the Gauss–Newton case or at the initial state as in Gradient descendent method are combined in the LMA for optimization^32,33^.

The input and target/output parameters for the ANN tool developed have been outlaid in supplementary table T2. Results from experimentation are used as initial training points and the training is carried out until the sum of the squared errors are minimized to < 0.001. The major goal here is to develop a system that can predict the best coating material, technique, number of coats and parameters for stable coatings on paper surfaces.

## Results and discussion

The viscosity and surface tension data obtained from the rheometer for the different solutions and ink are summarized in the Table 2. TEOS based solution and ink gelates to form hydrogel (in an airtight container) and xerogel (in open environment) as shown in supplementary Figure S1 in the time span of one week. In case of TMMS, the evaporation of ethanol, ammonia and DI water increases the viscosity and surface tension of the solution and the ink in a time span of around one week. Thus, only freshly prepared solution/ inks have been used for further studies. The print head of the inkjet printer is thoroughly washed after each cycle of printing using a specially realized wash buffer (containing 50% DI water + 50% ethanol absolute) to avoid cross-contamination and clogging problems.

**Table 2:** Viscosity and surface tension data for the TEOS and TMMS based solutions and inks freshly prepared and after 1 week.

Sample	Viscosity (Pa.s) @25 °C		Surface Tension (N/m) @25 °C		Fromm Number	
	As prepared	After 1 week	As prepared	After 1 week	As prepared	After 1 week
Ts1	0.0215	0.0637	33.25	45.72	2.568	2.063
Ts2	0.0367	Complete gelation	34.77	Complete gelation	3.083	NA
Ti1	0.0109	0.0599	30.59	37.64	6.125	2.641
Ti2	0.0114	Partial gelation	29.43	Partial gelation	7.258	NA

### X-ray diffraction (XRD)

**Figure 2:**
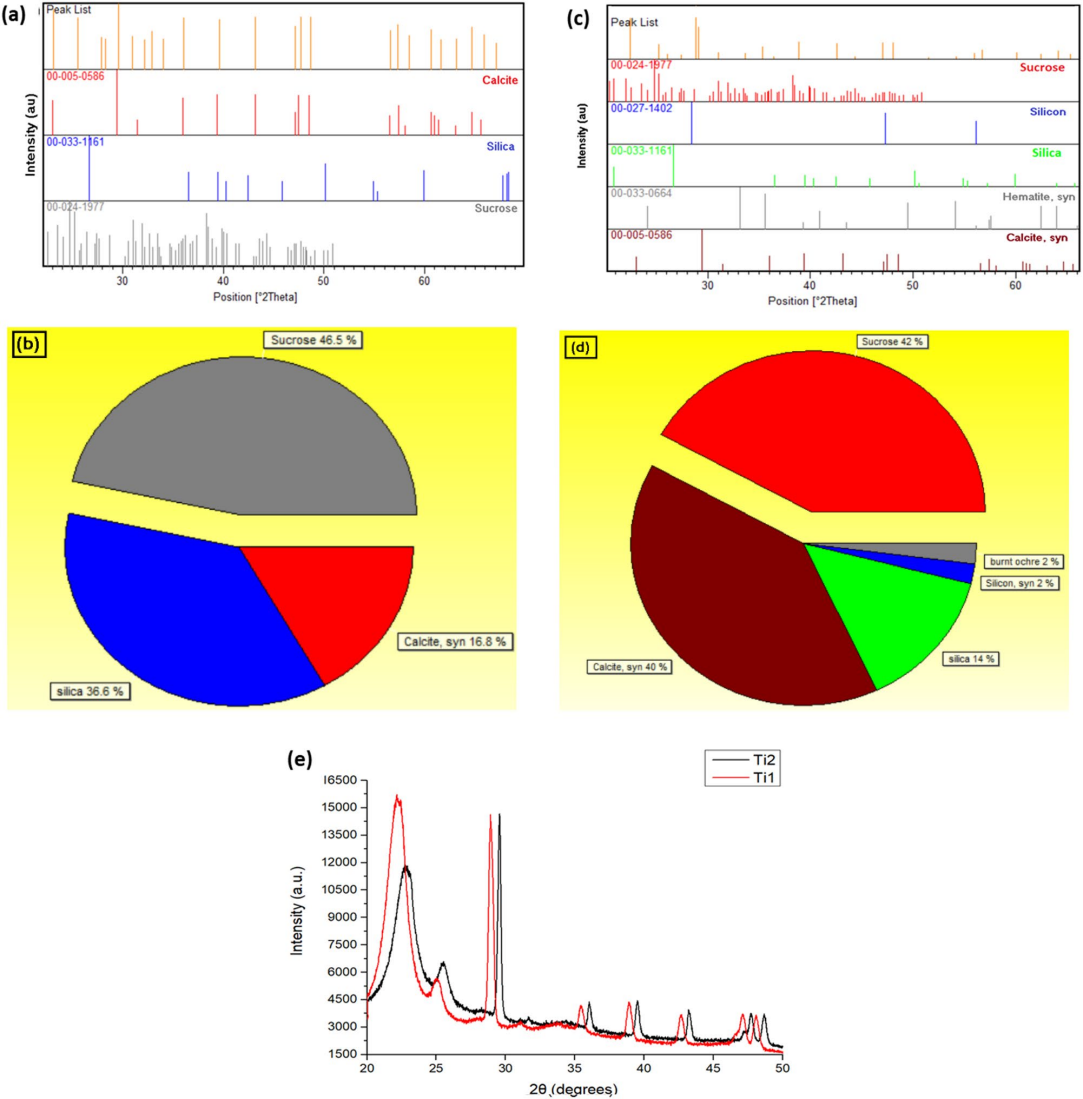
XRD peak analysis of: (**a**,**b**) TEOS and (**c**,**d**) TMMS dip coated paper respectively; (**e**) XRD intensity plot for paper dipped 5 times in Ti1 and Ti2. (Pan Analytical X’pert Powder Diffractometer/ X’Pert Highscore software).

Figure 2a,c show the XRD peak pattern of TEOS and TMMS dip coated paper samples respectively analysed for presence of different agents. The patterns show the presence of silica along with sucrose and calcite in case of TEOS coated samples (Fig. 2b) while in case of TMMS coating some silicon and hematite (burnt ochre) agents are also present as shown in Fig. 2d. The diffraction pattern of the coated samples shows the formation of new phases over and above the sucrose content of the substrate paper implying chemical bonding between the coated layers and the substrate leading to changes in hydrophobic properties. Figure 2e shows the basic XRD intensity curves for papers with multilayers of the solutions.

### Hydrophobicity due to TMMS and TEOS based agents on cellulosic paper

**Figure 3:**
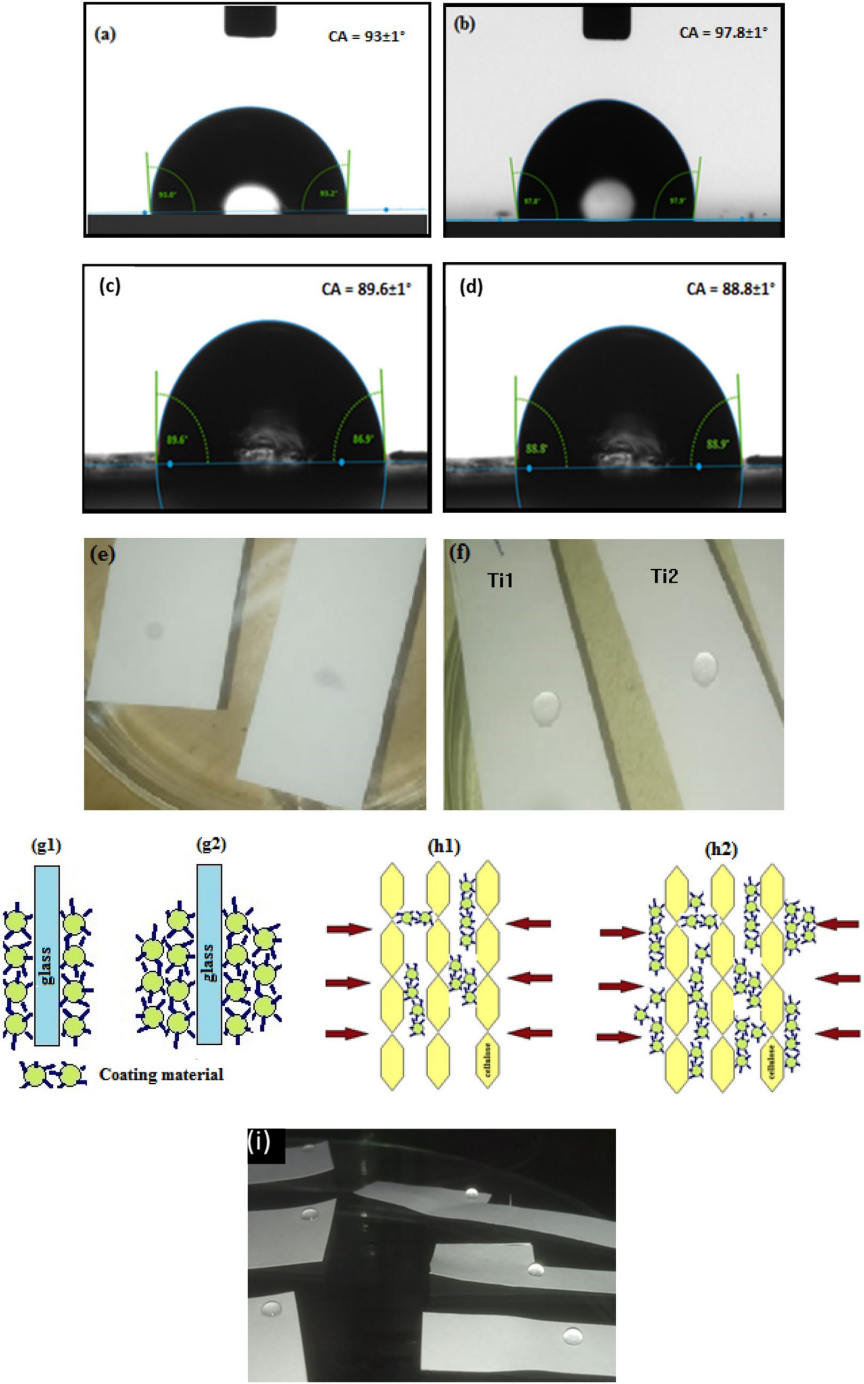
(**a**,**b**) Contact angle on Ts1 and Ts2 dip coated samples; (**c**,**d**) Contact angle on Ti1 and Ti2 dip coated samples (Contact angle goniometer (DATA Physics OCA 15 EC); (**e**) water droplet getting absorbed on uncoated paper; (**f**) water droplet on Ti1 and Ti2 single dip coated paper**; **(**g1**,**g2**) hydrophobic coating on surface of glass or metals; (**h1**,**h2**) hydrophobic coating on cellulosic paper; (**i**) water droplets on single and multiple dip coated paper samples in Ti1.

When a cellulosic paper is dipped in water it starts absorbing instantly. However, the contact angle changes from zero to some finite value after the dip coats. We found the contact angle of Ts1 coated paper to be 93° and the Ts2 coated paper as 97.8° respectively (Fig. 3a,b). Similarly, for coatings of ink Ti1 and Ti2, the coated paper strip showed contact angles of 89.6° and 88.8° respectively (Fig. 3c,d). The frame capture is done through the goniometer after the drop is allowed to stabilize for 10 secs. This indicates that both water retention and hydrophobic properties have been imparted to the paper after a single dip although the contact angle for the ink coating is seen to be lesser with respect to original solution due to the addition of other chemical agents. The contact angles have been found to be almost equal on both front and back of the paper after dip coating for all cases. All further experimentation has been carried out using Ti1 and Ti2 ink solutions. Figure 3e,f show how the uncoated and single dip coated substrates behave when water is dropped on the respective surfaces. It can be seen that the water droplets get easily absorbed on uncoated paper surfaces within seconds while it is retained on both Ti1 and Ti2 coated surfaces even after 10 min.

The major property of any paper is to absorb liquid due to their cellulosic fiber structure which is different from other surfaces like glass or metals. In case of glass or metals the agent gets coated on surface from the first dip and forms layers as time or number of coats increase (Fig. 3g1,g2) whereas when paper is dipped in the coating material, it first starts absorbing the agent (Fig. 3h1). As the time or number of coats increases, the gaps in cellulosic structure get filled and the agent starts getting coated on the surface (Fig. 3h2). Figure 3i shows water droplets on paper samples dipped once in Ti1 to the left which become more spherical as seen to the right when the paper is dipped multiple times (3 times) in the same solution. Thus, further studies are done to understand how various coating parameters for different coating techniques affect the hydrophobic nature of the paper surface.

### Coating techniques and parameter optimization

The various processes of dip, spin, spray and inkjet printing are studied using the different parametric values as in Table 2 shown earlier and the best combination of process parameters for both Ti1 and Ti2 is determined for each process using these experiments. These results will further be used to teach the machine learning algorithm to derive the best process alternatives.

#### Dip coating

**Figure 4:**
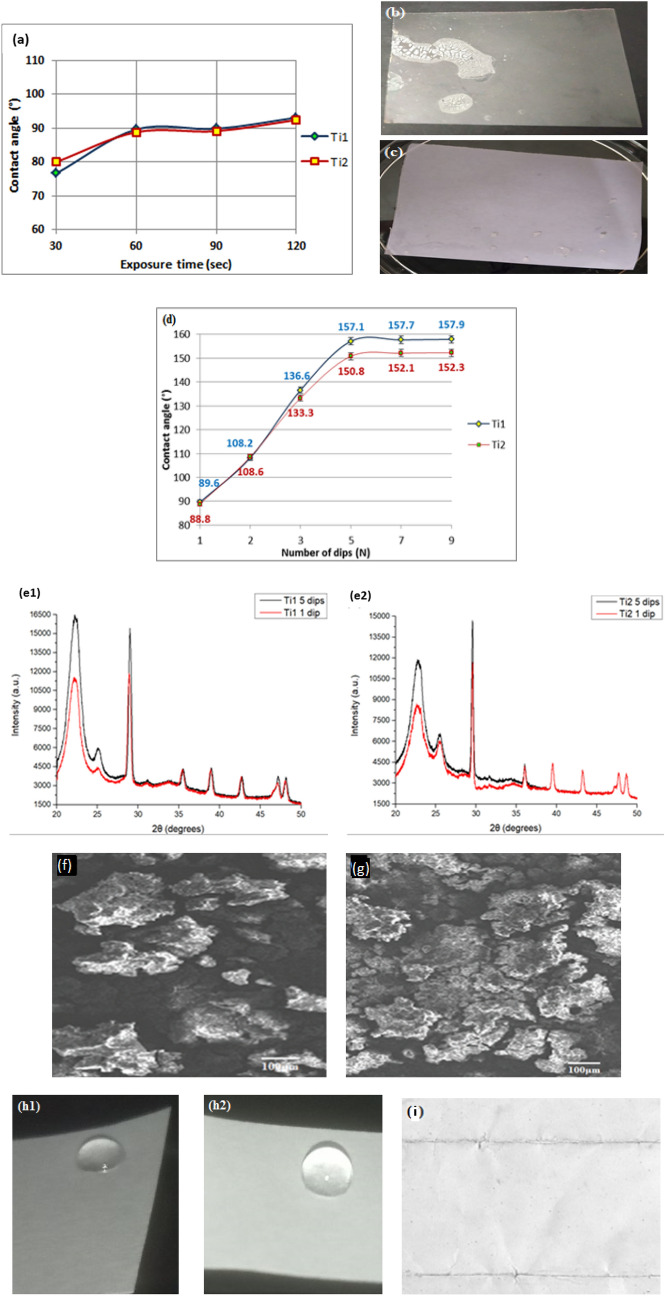
(**a**) Contact angle measurements at 30, 60, 90 and 120 secs for TMMS and TEOS dipped paper; (**b**) asperities on dried paper surface after 2 coats for 90 s dip coats, (**c**) surface asperities on 120 secs dipped paper after drying; (**d**) Number of dips versus the contact angle for Ti1 and Ti2 solutions; (**e1,e2**) XRD intensity curves for paper dipped once and 5 times in Ti1 and Ti2 respectively (Pan Analytical X’pert Powder Diffractometer/ X’Pert Highscore software); (**f,g**) SEM images of Ti2 coated surface after 1 dip and 5 dips respectively (EVO 50 (CARL Zeiss) system); (**h1**,**h2**) water droplet on paper dipped once and 2 times for 60 secs; (**i**) 9 times dip coated paper stiffening and showing wrinkles when folded.

In this module the coating time of the paper strips while using a dip coating process is varied for 30, 60, 90 and 120 secs for strips coated with Ti1 and Ti2 inks. The samples are vertically dipped and retracted at 1 mm/sec for uniformity of coating across all samples and dried at 25 °C for 30 min. The contact angle measurements for different dip time after drying are shown in Fig. 4a (single coat). It can be seen that for 60 and 90 s dips the maximum contact angle of around 89.6° and 89.8° respectively for coats of Ti1 and 88.8° and 89.1° comes for coats of Ti2. The surface started showing asperities in case of 90 s (Fig. 4b) after the second dip although no such problems were found in case of dips made for 60 s. Contact angle formed over the 30 s coat is found to be lower as the paper has very less exposure time to the agents while the paper strip coated for 120 s when dried develops surfaces with asperities and deterioration of paper as shown in Fig. 4c with slightly higher contact angle of 93.1° and 92.4° for Ti1 and Ti2 respectively. So, for all further experimentations dip coat of 60 secs with 30 min drying time between coats was used.

Results for the average contact angles with respect to number of layers of coating made on paper surfaces are shown in Fig. 4d. It is seen that as number of dip coats increase, the contact angle increases till 5 dips. After this a plateauing action is seen for further increase in number of dip cycles implying saturation on the coated surface. The contact angle increases with number of coating layers as more and more agents get deposited with each coating following which there is saturation and negligible changes in contact angle values. Figure 4e1,e2 shows XRD intensity rise and a slight positive shift in the angle (2θ) when the paper strip is dipped once and 5 times respectively in Ti1 and Ti2 implying better chemical bonding and crystalline structure with respect to coating layers. It also shows increase in the concentration of silica and calcite leading to higher hydrophobicity. Figure 4f,g show SEM images of the surface morphology after 1 and 5 dips respectively for the Ti2 coatings. The surface becomes superhydrophobic after 5 or more layers are deposited with either of the solutions. Similar trends are shown on both sides of the paper. Figure 4h1,h2 shows water droplets on paper dipped for 60 secs once and twice respectively. The paper becomes too stiff with a loss in flexibility beyond 7 layers. Figure 4i shows such an issue for paper dip coated 9 times in Ti2 getting creased when folded due to stiffening.

#### Spin coating

During spin coating, as soon as the ink solutions are dropped onto the surface, it is completely absorbed by the paper. Even after 5 cycles the paper keeps on absorbing the solutions and the process of spin coating cannot be carried out. So, it is concluded that spin coating is not a suitable technique for coating these TEOS or TMMS based solutions on paper.

#### Spray coating

For spray coating experiments, dispensing speed of 5 mL/min are used to check the changes in the coating properties for various raster speeds of 25, 50 and 75 mm/min in a straight-line manner on paper surface for both Ti1 and Ti2. The maximum contact angle of 83.5° and 85.1° for Ti1 and Ti2 respectively is obtained for raster speed of 50 mm/min while reduced to 76.3° and 77.9° for speed of 75 mm/min. When a speed of 25 mm/min is used, the dried samples caused either deterioration of the paper surface or developed asperities over it. This made the same unsuitable for further experimental use. Thus, for all further experiments a spray speed of 50 mm/min with a dispensing speed of 5 mL/min in a straight-line manner is used.

**Figure 5:**
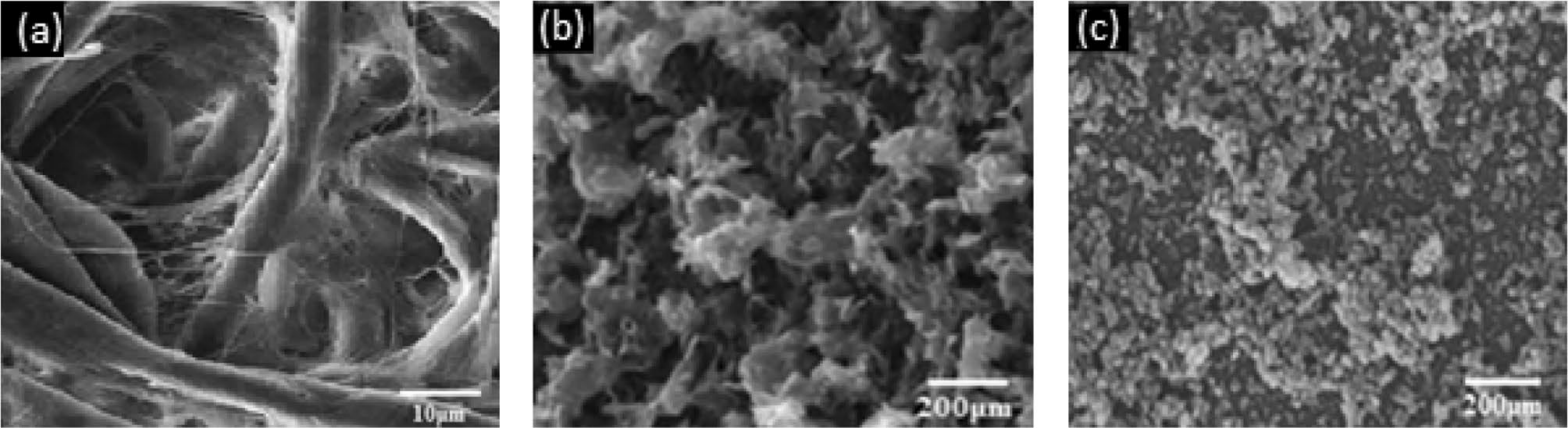
(**a**) SEM image of Uncoated cellulose paper; SEM image of paper spray coated with (**b**) Ti1 ink solution, (**c**) Ti2 ink solution. (EVO 50 (CARL Zeiss) system).

Further, a maximum contact angle of 134.2° and 138.7° is obtained for Ti1 and Ti2 respectively after 7 spray cycles after which the paper starts to deteriorate and crumble on drying. The contact angle on the non-sprayed side is found to be 118.4° and 120.9° respectively after 7 spray cycles. Figure 5a shows the SEM image of uncoated cellulosic paper while Fig. 5b,c shows the SEM image of Ti1 and Ti2 spray coated paper samples respectively showing the changes in the cellulosic structure of paper due to gelation in case of Ti1 and deposition and agglomeration of silica nanoparticles in case of Ti2. Cellulosic paper (Fig. 5a) absorbs water due to the empty structures inside the paper substrate which is filled in case of coating with either of the ink solutions with silica nanoparticles thereby making it hydrophobic.

#### Inkjet printing

**Figure 6:**
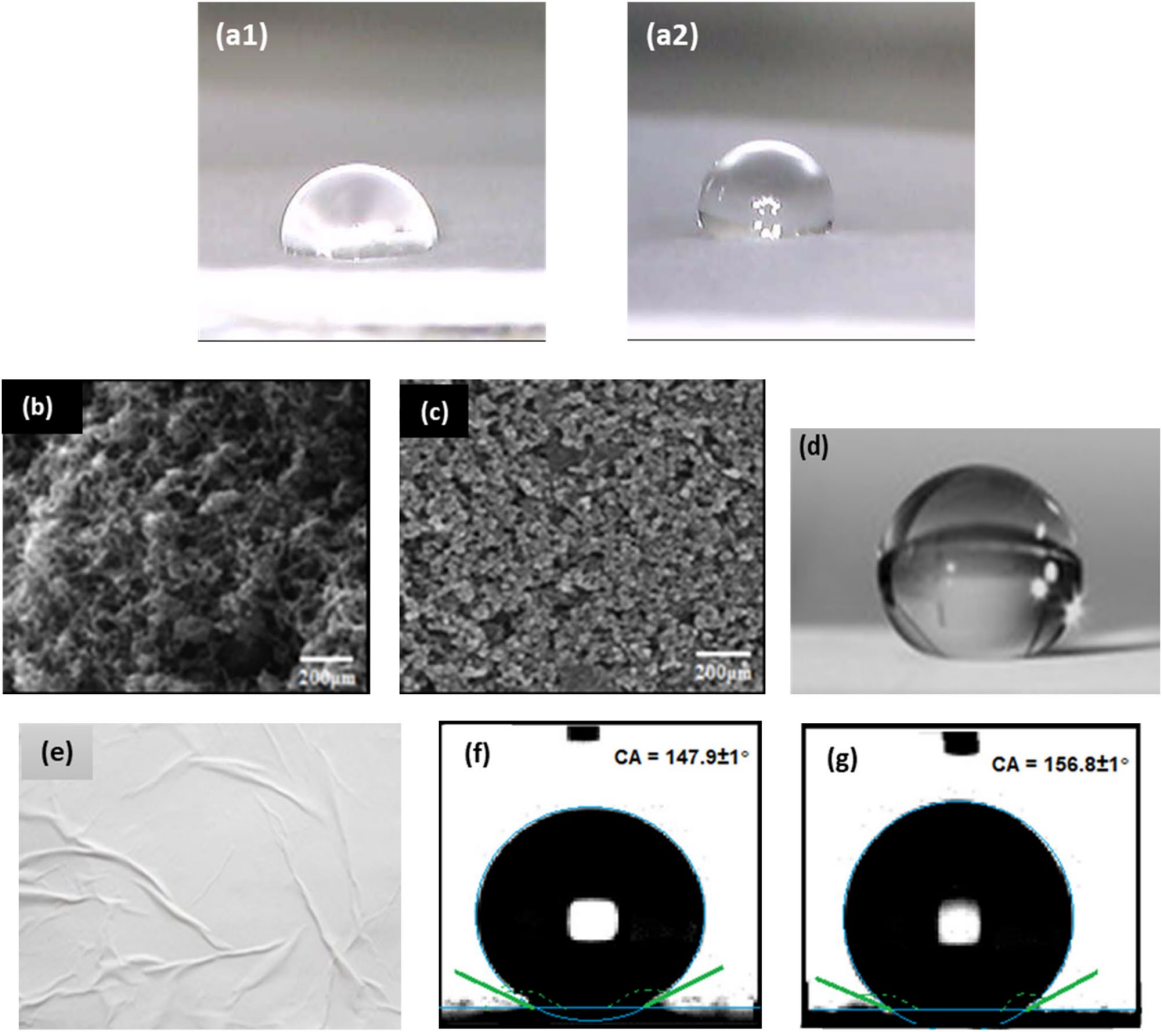
Water droplets on (**a1**) low quality and (**a2**) high-quality print areas after first print of Ti1; SEM images of paper spray coated with (**b**) Ti1 ink solution, (**c**) Ti2 ink solution (EVO 50 (CARL Zeiss) system); (**d**) Perfectly spherical water droplet on 7 times Ti1 printed paper; (**e**) Coated paper getting wrinkled and crumpling after the 7th cycle; Static contact angle on paper printed 5 times using (**f**) Ti1, (**g**) Ti2 (Contact angle goniometer (DATA Physics OCA 15 EC).

In these set of experiments paper substrate were quoted using low- and high-quality print modes of the EPSON printer. For the low-quality mode, contact angles of 72.5° and 75.8° and for high quality mode contact angles of 91.6° and 95.4° for single coat of Ti1 and Ti2 respectively were obtained. The difference in contact angles between the two modes is due to higher dots per inch improving the detail and resolution of the print thereby better spreading of the ink on the paper surface. Figure 6a shows the shape of water droplets on low quality and high-quality print areas after a single cycle of Ti1 and similar nature is seen for prints of Ti2. It can be seen that the water droplet is more spherical on the high-quality print side implying better hydrophobic property after the first print. Thus, for further experimentation the high-quality prints are used.

Papers printed using Ti1 and Ti2 based inks showed maximum contact angle of around 147.9 ± 1° and 156.8 ± 1° respectively after 5 printing cycles after which not much change was observed. The contact angles for the back side (non-printed side) are found to be around 73.2° and 73.8° respectively for single print which rise to 115.5° and 117.6° after 5 cycles. Figure 6b,c show the Sem images of the paper surface after 5 coats of Ti1 and Ti2 inks respectively. Perfectly spherical water droplets are observed on the 5 times coated paper surface as shown in Fig. 6d which indicates that the surface is superhydrophobic. Stiffening and wrinkling is observed in the printed areas when dried after 7 cycles as shown in Fig. 6e in both the cases. Figure 6f,g shows the contact angle on paper printed 5 times with Ti1 and Ti2 respectively.

### Process based comparisons

The relative comparison of contact angle for 7 cycles of each of the 3 techniques, dip, spray and inkjet print on both the front (main coated side) and the back sides for Ti1 andTi2 are shown in supplementary figure S3. In case of dip coating both front and back side get coated while for case of spray coating and printing the backside remains the uncoated surface and hence showing lesser contact angles. It can be seen that Ti1 ink shows best contact angle results with dip coating technique for 7 cycles while Ti2 shows better results when printed on the paper surface.

### ML training and pilot study

**Figure 7:**
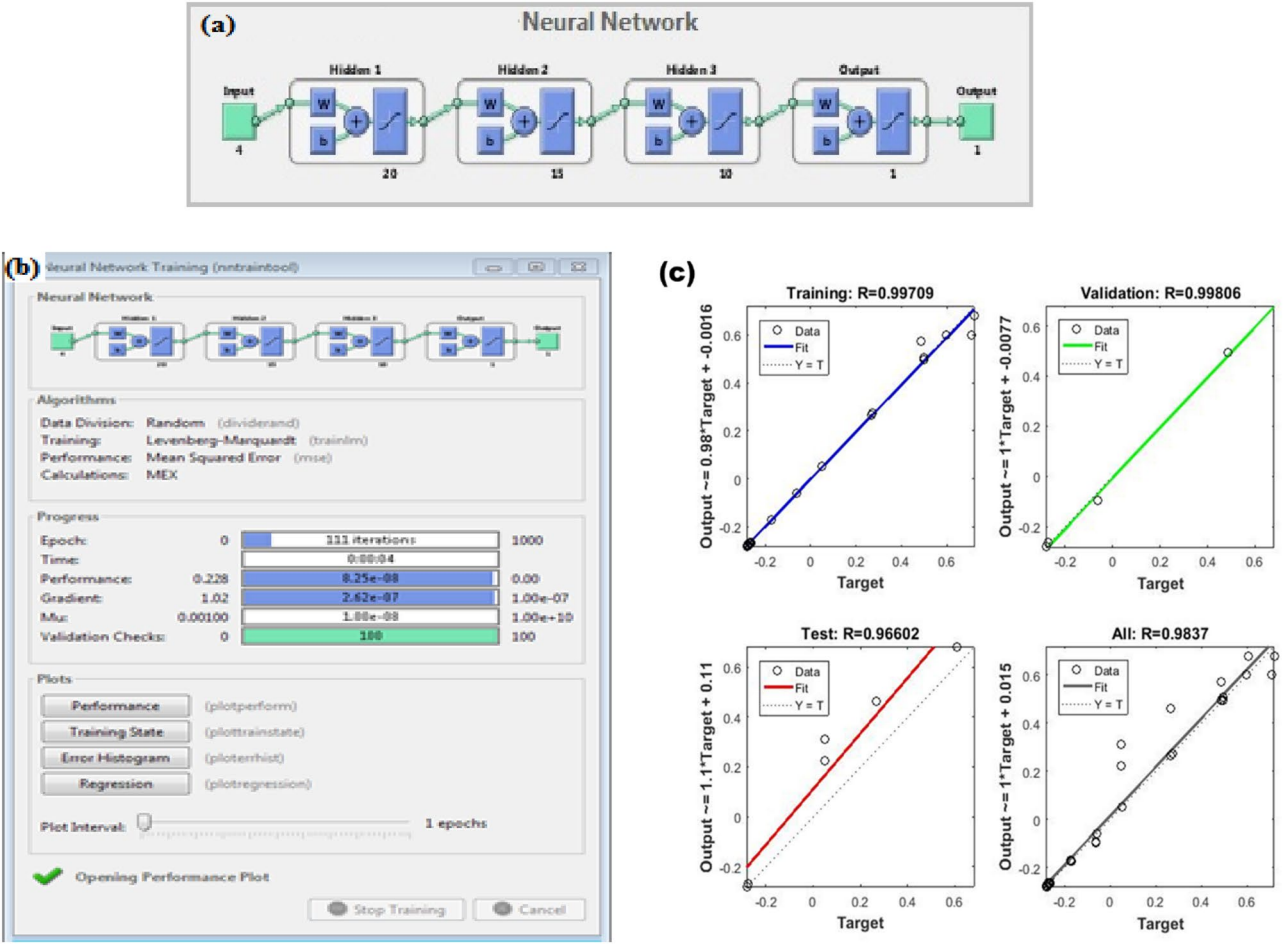
(**a**) Neural network generated for training, (**b**) training the network, (**c**) regression plots after 5 training cycles for training, validation and testing of the network showing convergence (R≈1) for number of cycles. (MATLAB R2016a).

The results from all the experiments using Ti1 and Ti2 as coating materials and dip, spray and printing as coating techniques on cellulosic paper are used to develop the trained neural network on Matlab. 42 data points are used for training initially and the input (base material, initial contact angle, final contact angle front side and back side) and output/ target data points (coating material, coating technique and number of coats) are used. The neural network consists of 3 hidden layers of neurons using tangent sigmoid (tansig) as the transfer function with Levenberg–Marquardt algorithm (LMA) used as the optimization technique as shown in Fig. 7a. Coating material, coating technique and the number of coats are all integer values. The training input with targets is given in Section S4 while the detailed code and the output after running the code in Matlab is provided in the section S1 of the supplementary material.

The developed network is trained for upto 5 cycles until convergence (R≈1). Figure 7b shows the training of the network and Fig. 7c shows the convergence of all parameters after 5 cycles of training.

This ML based network is then further used for predicting the best technique and parameters for given set of input parameters as shown in Table 3 in the pilot study. It is found that the value of contact angle obtained using the parameters predicted using the trained network are almost similar to the required values with a maximum error of ± 4.5°.

**Table 3:** Input and predicted output parameters from ML system, the experimental results obtained using the predicted parameters and the error.

Sample input				Predicted output parameters			Experimental result			Error	
M	IC	FCF	FCB	CM	CT	N	IC	FCF	FCB	FCF	FCB
1	0	0	0	1	1	0	0	0	0	0	0
1	0	85	85	1	1	1	0	88.6	84.9	3.6	− 0.1
1	0	120	95	1	3	3	0	116.4	92.1	− 3.6	− 2.9
1	0	140	140	1	1	4	0	144.5	143.9	4.5	3.9
1	0	155	115	2	3	5	0	156.8	112.6	1.8	− 2.4

Further studies on different base materials, coating materials etc. can be incorporated into this ML data base and can be used in future to predict the best coating technique for given set of conditions. The ML system becomes more and more reliable as the number of training points increase.

## Conclusion

The current experimentation primarily studies the use of ML for predicting the process parameters to develop hydrophobic surfaces on paper using different coating materials and coating techniques. The ML system is trained by doing experimentation using both silane based and sol–gel based solutions and using different coating techniques like dip, spin, spray and inkjet printing. From experimentation it is concluded that the number of coats and the type of coating technique influences coating of different materials onto the surface. Dip coating technique is found suitable for TMMS based silane ink solutions whereas inkjet printing is found as a better technique for coating TEOS based sol–gel inks on paper substrates. After certain number of cycles (5 cycles in most cases) the change in contact angle is low or the paper starts to deteriorate. Also freshly prepared ink solutions are to be used as the properties of the solutions change with time on keeping. The ML based trained system can predict the best set of process parameters to prepare a hydrophobic surface of our requirement. The reliability of this system will further improve on adding more and more data points.

The experimental set can further be expanded for other substrates like glass, metals etc. or other coating materials and the same can be added to the training set of the ML code for precise prediction according to the needs of the customer.

## Supplementary Information

Supplementary Information.
