# Supplementary material for: Development of hydrophobic paper substrates using silane and sol–gel based processes and deriving the best coating technique using machine learning strategies
Source: Sci Rep. 2021 May 31;11:11352. doi: 10.1038/s41598-021-90855-7 (PMC8167096; doi:10.1038/s41598-021-90855-7)
Supplement: Supplementary file 1 — Supplementary Information. [file 41598_2021_90855_MOESM1_ESM.docx]

**Development of Hydrophobic Paper substrates using Silane and Sol-gel based processes and Deriving the Best Coating Technique using Machine Learning Strategies**

Kapil Manoharan^1^, Mohd. Tahir Anwar ^2^, Shantanu Bhattacharya^1#^

1. Microsystems Fabrication Lab, Department of Mechanical Engineering, Indian Institute of Technology Kanpur
2. BNCET, Lucknow

# Corresponding Author: bhattacs@iitk.ac.in

**Supplementary Information:**

**Figure S1:** *Ti2 after 1 week*

**(a)** xerogel in open environment, **(b)** hydrogel in air tight container.


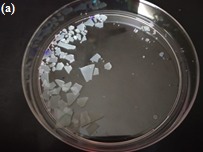

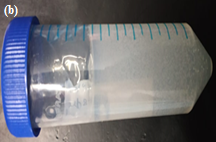


**Figure S2:** *Ink formulation process*

**(a, b)** TEOS based sol-gel solution/ink


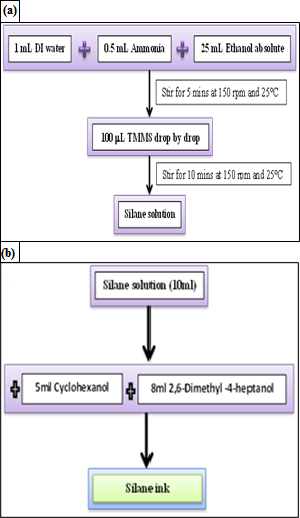


**(c, d)** TMMS based silane solution/ink


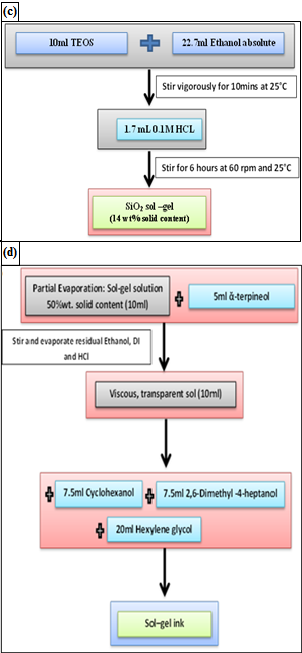


**Figure S3:** *Comparison of contact angle for dip coated, spray coated and printed paper surface:*

**(a)** Ti1 ink on front side; **(b)** Ti2 ink on front side


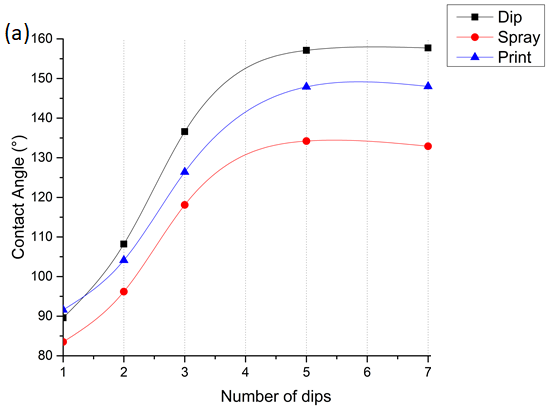

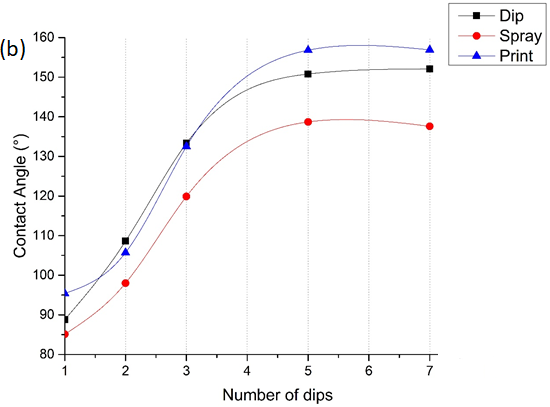


**(c)** Ti1 ink on back side; **(d)** Ti2 ink on back side


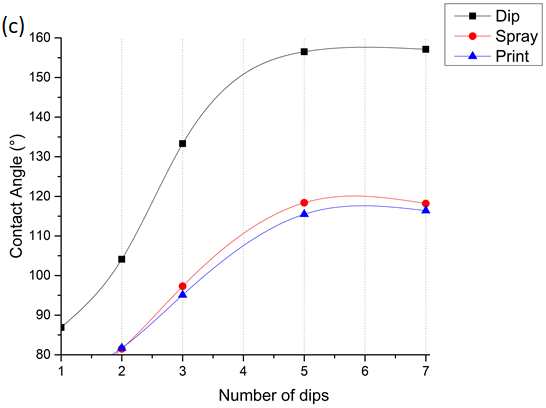

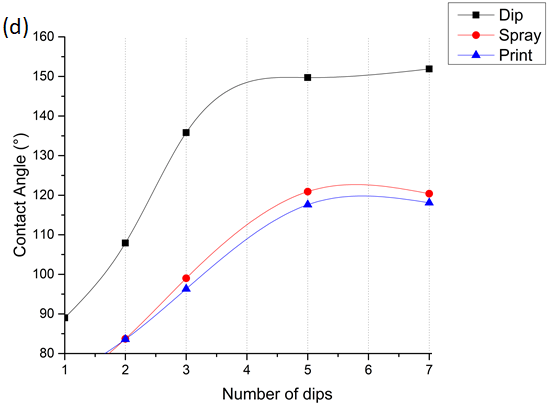


**Table T1:** Process wise classification of different parameters, substrates and coating material requirements.

| **Coating Process** | **Parameters** | **Substrates** | **Coating materials** |
| --- | --- | --- | --- |
| **Dip coating** | Viscosity and surface tension of solution, dip time, retraction speed, dip angle, substrate size. | Rigid surfaces: glass, metals etc.  Flexible surfaces: paper, plastic films etc. only when using solutions with low viscosity and surface tension. | Wide range of solutions can be used such as silanes, siloxanes, sols, silica-based epoxy resins etc. |
| **Spin coating** | Viscosity and surface tension of solution, spin speed and time, substrate size and porosity/ absorptivity. | Wide range of substrates like glass, metals, plastic films but not suitable for textiles and other super flexible substrates which have high porosity/ absorptivity. | Same as dip coating. |
| **Spray coating** | Viscosity and surface tension of solution, spray speed, spraying method. | Can be used for all kind of surfaces/ substrates | Same as dip coating with exception of solutions having high viscosity. |
| **Inkjet Printing** | Rheology of the ink, resolution of print, type of printing technique/ printer used. | Mostly used on flexible substrates like plastic films, canvas, paper etc. | Solutions or inks of silanes, siloxanes and silica-based agents meeting the specific rheology requirements. |
| The number of coats, drying time, drying temperature are dependent on the material to be coated while the thickness of the coated layer depends on several parameters according to the process parameters and material used for coating. | | | |

**Table T2:** Input and output/target parameters with actual and designated values for the training process.


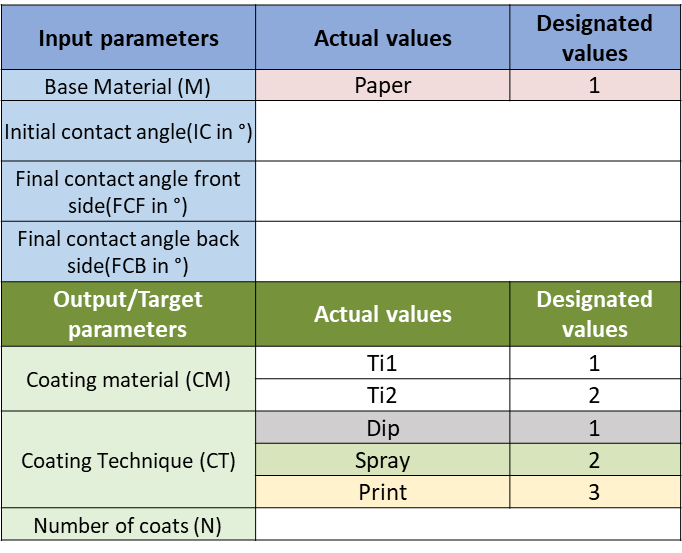


**S1:** *Matlab code and Output*

***Matlab Code***

**%reading excel file**

t=xlsread('SHS');

**%Inputs**

X=t(:,1:4)';

%Targets

T1_d=t(:,9)';

T1=int8(T1_d);

T2_d=t(:,10)';

T2=int8(T2_d);

T3_d=t(:,11)';

T3=int8(T3_d);

**%Neural Network creation and training:**

**%coating material**

net1= feedforwardnet([20 15 10]);

net1.performFcn='mse';

net1.layers{1}.transferFcn = 'tansig';

net1.layers{2}.transferFcn = 'tansig';

net1.layers{3}.transferFcn = 'tansig';

net1.trainParam.max_fail=100;

net1.trainParam.epochs=1000;

net1=train(net1,X,T1);

**%coating technique**

net2= feedforwardnet([20 15 10]);

net2.performFcn='mse';

net2.layers{1}.transferFcn = 'tansig';

net2.layers{2}.transferFcn = 'tansig';

net2.layers{3}.transferFcn = 'tansig';

net2.trainParam.max_fail=100;

net2.trainParam.epochs=1000;

net2=train(net2,X,T2);

**%number of cycles**

net3= feedforwardnet([20 15 10]);

net3.performFcn='mse';

net3.layers{1}.transferFcn = 'tansig';

net3.layers{2}.transferFcn = 'tansig';

net3.layers{3}.transferFcn = 'tansig';

net3.trainParam.max_fail=100;

net3.trainParam.epochs=1000;

net3=train(net3,X,T3);

**%checking : deriving predicted values:**

CM= sim(net1,X(:,19:23));

CT= sim(net2,X(:,19:23));

N= sim(net3,X(:,19:23));

**%displaying normal original vs predicted values:**

A1=[T1(1,19:23); CM]

B1=[T2(1,19:23); CT]

C1=[T3(1,19:23); N]

**%sample input (after 5 cycles training)**

Z=t(:,5:8)';

%deriving predicted values:

Y1= int8(sim(net1,Z(:,1:4)));

Y2= int8(sim(net2,Z(:,1:4)));

Y3= int8(sim(net3,Z(:,1:4)));

**%displaying sample data predicted values:**

[Y1]

[Y2]

[Y3]

***Matlab Output***

1. *Output- original vs predicted values:*

After Training round 1:

A1 =

1 1 1 2 2

1 1 1 2 2

B1 =

3 3 3 1 1

3 3 3 2 1

C1 =

5 7 8 0 1

5 7 7 0 1

After Training round 5:

A1 =

1 1 1 2 2

1 1 1 2 2

B1 =

3 3 3 1 1

3 3 3 1 1

C1 =

5 7 8 0 1

5 7 8 0 1

1. *Output- Sample data predicted values:*

Y1 =

1 1 1 1 2

Y2 =

1 1 3 1 3

Y3 =

0 1 3 4 5
